# Do you feel your body right now? Ecological momentary assessment of interoceptive intensity and valence across multiple domains in everyday life

**DOI:** 10.3389/fpsyg.2026.1930018

**Published:** 2026-10-14

**Authors:** Christian Rominger, Marta Walentynowicz, Olga Pollatos, Christine Marie Schick, Andreas R. Schwerdtfeger

**Affiliations:** 1 Institute of Psychology, University of Hildesheim, Hildesheim, Germany; 2 Dornsife Center for Self-Report Science and Center for Economic and Social Research, University of Southern California, Los Angeles, CA, United States; 3 Institute of Psychology and Education, Ulm University, Ulm, Germany; 4 Institute of Psychology, University of Graz, Graz, Austria

**Keywords:** affect, bodily sensations, body awareness, EMA, interoceptive sensibility

## Abstract

Researchers usually assess interoception (i.e., the perception of inner bodily states) under laboratory conditions, once, with a focus on one organ, mostly the heart. The present study expands this research and presents findings from two independent studies, demonstrating the reliability and validity of an ecological momentary assessment (EMA) approach to study interoception in different modalities at the very same time from a subjective perspective. We asked more than 250 participants to rate how intensely they feel their heart, gut, and breath at random moments in everyday life, as a measure of interoceptive attention. We further assessed the appraisal of these interoceptive body sensations by means of valence ratings. Across two samples (N1 = 141, N2 = 112), we demonstrated high reliability of this assessment at the between-person level and modest reliability at the within-person level. We further examined convergent and divergent validity for both subjective measures. The study findings highlight promising psychometric properties and applicability of this novel assessment of ambulatory interoception. This method enables large-scale field studies to investigate interoceptive attention and appraisal on a momentary basis to understand the role of bodily sensations in daily functioning and health.

## Introduction

Interoception, defined as the perception of internal bodily signals, is relevant for health and well-being (Khalsa et al., 2018). Perceiving bodily signals informs us about emotional states and allows an adaptation to the needs of the organism in an ever-changing environment (Petzschner et al., 2021). Greater access to one’s own bodily sensations may allow better regulation of emotions and thereby promote health and well-being in the long run (Brewer et al., 2021; Ibanez and Northoff, 2024; Khalsa et al., 2018; Nord and Garfinkel, 2022; Zamariola et al., 2019). Recently, the need for a stronger situational and contextual focus on interoception has been voiced (Rominger and Schwerdtfeger, 2023; Murphy, 2022; Desdentado et al., 2026). However, the ambulatory tools to assess and study interoception in everyday life situations are still limited (Lopez-Martin et al., 2025), which restricts the ecological validity of study findings and their generalizability to real world applications.

Interoception research commonly distinguishes between interoceptive accuracy, interoceptive sensibility, and interoceptive metacognition (Garfinkel et al., 2015). Interoceptive accuracy is the absolute correspondence between perceived and objectively recorded signals from the inner body, such as the heart rate. Interoceptive sensibility means self-perceived tendencies in interoceptive sensations (e.g., confidence ratings, intensity ratings, etc.). Metacognition refers to the correspondence between both interoceptive accuracy of sensations and the confidence in these sensations, that is, knowing when one is perceiving bodily signals more or less accurately (for further dimensions of interoception see, e.g., Murphy et al., 2019; Suksasilp and Garfinkel, 2022).

Given these three basic dimensions, the main limitations of existing research are the following: First, the vast majority of studies investigating interoceptive sensibility or interoceptive accuracy are laboratory studies (though there is ambulatory research available; Rominger and Schwerdtfeger, 2023; Schwerdtfeger and Rominger, 2024a; Höller et al., 2021; Ponzo et al., 2021; MacVittie et al., 2025; Poerio et al., 2024). This reduces the ecological validity of interoception assessments and the resulting reports. Second, laboratory studies limit the possibility to scale up study designs. Most studies rely on smaller and less diverse (often academic) samples. Third, most of these laboratory studies assessed interoception once, conceptualizing interoception as an invariant trait, which ignores within-person fluctuations and contextual changes (see, e.g., Wittkamp et al., 2018; MacVittie et al., 2025; Desdentado et al., 2026). Fourth, the majority of studies assessing interoceptive accuracy focused on only one organ, mostly the heart (and occasionally breathing and gastric functions, Harrison et al., 2021; Herbert et al., 2012) and often assume that the observed effects generalize from one domain to the other, which has been repeatedly questioned (see, e.g., Ferentzi et al., 2018). Fifth, retrospective interpretations and subjective experiences might more strongly influence global trait questionnaires on interoceptive sensibility, thus challenging validity (Mehling et al., 2018; Desmedt et al., 2022; Vlemincx et al., 2023; Vig et al., 2022).

Furthermore, in addition to the dimensions of Garfinkel et al. (2015) interoceptive theories emphasize the necessity of further subdividing interoception into distinct psychological processes, such as attention (the degree to which bodily signals are monitored) and appraisal (the affective evaluation of those signals; Farb et al., 2015; Khalsa et al., 2018; Murphy et al., 2020). Using two independent samples, the present study introduces two novel measures designed to assess this critical distinction in daily life, which might overcome some of the major interoception-specific research limitations discussed above. First, to assess interoceptive attention we applied the Momentary Interoceptive Intensity Scale (MIIS; items from Walentynowicz et al., 2023; see MacVittie et al., 2025 for one general awareness item and Poerio et al., 2024 for interoceptive attention items). This novel ecological momentary assessment (EMA) questionnaire asks participants at random moments in their daily lives about the intensity of momentary perception of bodily signals from three interoceptive domains (i.e., heart, gut, breath). This allows a more direct and ecologically valid assessment of self-reported attention to interoceptive body sensations (Suksasilp and Garfinkel, 2022; Murphy et al., 2020). The core idea of the ambulatory measure is that assessing judgments of the intensity of bodily signals in everyday life is a direct way to assess interindividual variations in interoceptive attention. For example, asking participants, after a run or while lying in bed, how strongly they perceive their body provides direct access to fluctuations in interoceptive attention that cannot be achieved in laboratory settings or through retrospective questionnaires, thereby highlighting the ecological validity of this assessment approach. By repeatedly administrating the same MIIS items, we can differentiate between-person (trait) and within-person (state) variation of interoceptive body sensations, an approach which has been advocated for recently (Rominger and Schwerdtfeger, 2023; Suksasilp and Garfinkel, 2022). The suggested ambulatory approach has the potential to overcome existing limitations and enable the assessment of multiple domains at multiple time points in diverse contexts in everyday life, thus elucidating when and under which conditions bodily signals are perceived.

Second, to assess interoceptive appraisal, we developed the Momentary Interoceptive Valence Scale (MIVS) which asks for the affective valence assigned to interoceptive sensations. By separating intensity from valence, we can disentangle the mere detection of bodily states (i.e., attention to) from the emotional meaning (i.e., appraisal) individuals ascribe to them. The MIVS asks participants about the perceived valence of sensations from the heart, the gut, and the breath (i.e., how positively/negatively the bodily signals are appraised in a given moment). Examining the valence of interoceptive sensations converges with recent suggestions to account for interoceptive interpretation and appraisal to further elucidate interoceptive awareness (e.g., Desmedt et al., 2025; Farb et al., 2015). Specifically, perceiving bodily signals negatively or positively could be more important for an individual’s well-being and psychosomatic symptoms than the mere intensity of the perception.

Taken together, study 1 aimed to (1) evaluate the reliability of the MIIS across multiple time points and interoceptive domains as a measure of interoceptive attention, and (2) provide initial evidence for its validity, including associations with valence ratings of bodily sensations by means of the novel Momentary Interoceptive Valence Scale (MIVS) as a measure of interoceptive appraisal. Importantly, in order to substantiate validity of the assessment, we aimed to analyze associations with different trait-related variables that have been related to interoception such as perceived stress (Schulz and Vögele, 2015; Schulz et al., 2020), alexithymia (Zamariola et al., 2018), psychosomatic symptoms (Ciaramella et al., 2023), happiness (Ferentzi et al., 2019), mindfulness (Hanley et al., 2017), intuitive eating (Poovey et al., 2022), body image (Todd et al., 2020), physical activity (Georgiou et al., 2015), and affect (Poerio et al., 2024).

## Methods

### Participants

The ethical committee of the University of Graz approved this study (GZ. 39/82/63 ex 2014/15), in which 167 participants took part. Responses completed in under 0.5 min and longer than 10 min were excluded. Furthermore, to ensure stable estimates, only participants who provided more than 10 valid prompts after applying these duration filters were taken for analysis. This resulted in a final analytical sample of 141 participants providing 5,913 valid prompts. Since the simulation study of Arend and Schäfer (2019) showed that detecting a small within-person effect with 80% power (significance level of 0.05) typically requires approximately 100 participants providing 20–30 measurement occasions, the current sample size seems sufficient. The mean age of the sample (102 women, 37 men, 2 diverse) was 25.82 years (SD = 9.05). All participants gave written informed consent prior to the assessment days in the Esmira app (Lewetz and Stieger, 2023) running on a local server at the university. Participants received course credits for their participation. Data collection was completely anonymized.

### Procedure and study design

The EMA took place for 7 days, starting at a random day. The Esmira app delivered 12 random prompts per day (09:00 a.m. - 09:00 p.m.). The minimum distance between prompts was 30 min. Participants could delay the prompts if wished for five times with 25 min in total. There were no time limits when answering a prompt. At the beginning of the study, all participants got written instructions and were asked to fill out questionnaires and demographic data. In case of any problem participants could communicate via a chat function implemented in the Esmira app with the research team. The study was conducted from 15.12.2023 till 10.01.2024. As compensation, participants received course credits if needed.

### Materials of the ecological momentary assessment

#### Momentary interoceptive intensity scale

The intensity of interoceptive sensations was assessed using the items of Walentynowicz et al. (2023). The MIIS measures interoceptive attention based on the subjectively perceived intensity of sensations from the heart (“at this moment, I feel my heartbeat”), the breath (“at this moment, I feel my breathing”), and the stomach/gut (“at this moment, I feel my stomach/gut”). Participants rated their interoceptive sensations using a visual analog scale ranging from 1 to 100. A score of 1 represents the minimum intensity, while a score of 100 represents the maximum intensity. For each interoception measurement, the three items were presented in randomized order. The overall score for interoceptive attention was calculated based on the aggregate mean across all three items (*M* = 37.97, SD = 17.68).

#### Momentary interoceptive valence scale

Additionally, we assessed the appraisal of the bodily sensations via valence ratings. Participants rated the items on a scale from 1 (unpleasant) to 100 (pleasant). We measured interoceptive valence of sensations of the heart (“at this moment, my heartbeat feels …”), breath (“at this moment, my breathing feels …”), and stomach/gut (“at this moment, my stomach/gut feels …”). For analysis, the mean across all three items per prompt served as an overall score for interoceptive valence (*M* = 67.49, SD = 18.32).

We conceptualized both scales, the MIIS and MIVS, as broad composite indices of generalized interoceptive attention and valence/appraisal, rather than strictly uni-dimensional latent traits. While distinct visceral systems (cardiac, respiratory, gastrointestinal) naturally exhibit unique contextual variance in everyday life, capturing a generalized interoceptive state requires aggregating across multiple primary domains (Vlemincx et al., 2023). The aggregation should ensure that the scale captures systemic bodily attention/appraisal rather than isolated somatic symptoms (see Supplementary Tables 1, 2, illustrating the inter-item associations at both the within-person and between-person levels). Robust between-person inter-item correlations support a generalized tendency for somatic focus, while more modest positive within-person correlations reflect the expected momentary fluctuations between localized bodily states.

### Additional momentary variables to investigate validity of the MIIS and MIVS

#### Momentary affect

To investigate within-person validity of interoceptive intensity and valence, we assessed current affective well-being with the Self-Assessment Manikins (SAM; Bradley and Lang, 1994), because research suggests a close link between both affect and interoception (Poerio et al., 2024; MacVittie et al., 2025). The SAM questionnaire provides a language-free method to capture participants’ affective states and consists of three figural items: pleasure, arousal, and dominance. Each item consists of five images that represent different intensities (representing a score-range from 1 to 5). The three scales were adapted for ambulatory data collection and multiple daily assessments, focusing on the participants’ momentary feelings at the prompt. The mean for pleasure was 3.75 (SD = 0.91), for arousal was 2.49 (SD = 1.04; with higher scores indicating higher arousal), and for dominance was 2.63 (SD = 0.95; with higher scores indicating higher dominance).

#### Hunger

Hunger is an interoceptive sensation (Nord and Garfinkel, 2022; Craig, 2002; Vaitl, 1996) and therefore well suited to serve as a validity measure at the state level (see, e.g., Rominger and Schwerdtfeger, 2023). We assessed perceived hunger at the moment of each prompt by means of a visual scale from 1 to 100 (“not hungry” to “very hungry”; *M* = 23.82, SD = 24.60).

#### Pain intensity

Similarly, since pain is related to interoceptive processing, depending on the origin (e.g., stemming from the viscera, also pain counts as an interoceptive sensation Nord and Garfinkel, 2022; Craig, 2002), it is well suited to serve as a validity measure at the state level. We assessed the perceived pain at the very prompt (“Are you currently experiencing physical pain? If so, how severe is it?”) by means of a visual scale from 1 to 100 (“no pain” to “extraordinarily strong pain”; *M* = 9.93, SD = 13.48).

#### Additional contextual information

At each of the 5,913 available prompts, participants indicated whether they were interacting with other people (2,340 prompts) or were alone (3,573 prompts). Participants further answered a question about their current location such as being at home (3,942 prompts) or somewhere else (1,971 prompts).

### Between-person variables assessed via questionnaire

#### Body appreciation scale

Positive body image was assessed using the German version of the Body Appreciation Scale-2 (BAS-2; Behrend and Warschburger, 2022), which measures appreciation for one’s own body. The 10 items of the questionnaire (e.g., “I respect my body”) were rated on a five-point Likert scale from 1 (*never*) to 5 (*always*). The internal consistency was α = 0.93 and mean score was *M* = 36.26 (SD = 7.72). This scale showed positive associations with interoceptive sensations in previous research (Todd et al., 2020).

#### Five facet mindfulness scale

We used the 39 items of the German version (Michalak et al., 2016) of the Five Facet Mindfulness Questionnaire (FFMQ; Baer et al., 2008). *Nonjudging* of inner experience refers to adopting a non-evaluative attitude toward current experiences (*α* = 0.89; *M* = 26.73, SD = 7.64). *Describing* is the ability to bring awareness to mental content using individual words or short phrases (*α* = 0.91; *M* = 28.19, SD = 6.86). *Observing* describes self-assessment regarding the ability to mindfully perceive a variety of external and internal stimuli (e.g., bodily sensations or emotions; *α* = 0.78; *M* = 28.31, SD = 5.71). *Acting* with awareness refers to the ability to fully engage in activities (*α* = 0.84; *M* = 25.40, SD = 6.28). *Nonreactivity* is the ability to refrain from reacting to events of inner experience (*α* = 0.84; *M* = 21.96, SD = 5.40). Participants rated the items on a 5-point Likert scale. We also calculated the total sum score of the FFMQ with *M* = 130.60 (SD = 21.38; *α* = 0.90). The FFMQ subscales showed positive associations with questionnaires assessing interoceptive awareness (i.e., MAIA) in previous studies (e.g., Mehling et al., 2012; Hanley et al., 2017).

#### Intuitive eating scale

To assess intuitive eating behavior, we used the German version of the IES-2 (Ruzanska and Warschburger, 2017). The questionnaire consists of 23 items, which allow to assess four subscales: Unconditional Permission to Eat (UPE; *α* = 0.72), Eating for Physical rather than Emotional Reasons (EPR, *α* = 0.88), Reliance on Hunger and Satiety Cues (RHSC, *α* = 0.93), and Body-Food Choice Congruence (B-FCC, *α* = 0.85). Responses were rated on a 5-point Likert scale from 1 (strongly disagree) to 5 (strongly agree). The total score α was 0.89 (*M* = 3.66, SD = 0.63). We included this scale because intuitive eating showed positive relationships with interoceptive sensibility (i.e., MAIA subscale, Poovey et al., 2022).

#### Subjective happiness scale

Since interoceptive sensibility was associated with happiness and subjective well-being (Ferentzi et al., 2019) we assessed general happiness in this study. We used the German version of the Subjective Happiness Scale (SHS; Lyubomirsky and Lepper, 1999). The SHS consists of a 7-point Likert scale with four questions. It demonstrates good internal consistency, with *α* = 0.88 (*M* = 4.92, SD = 1.22).

#### Trait emotional intelligence questionnaire short form

We used the German short form (Laborde et al., 2016) of the Trait Emotional Intelligence Questionnaire (TEIQue; Petrides and Furnham, 2003; Petrides, 2009; for a German version see Freudenthaler et al., 2008). The TEIQue-SF assesses global emotional intelligence as well as four subcategories: well-being, self-control, emotionality, and sociability. The questionnaire consists of 30 items (26 items forming the subcategories and 4 additional items; e.g., “In general, I am able to adapt well to a new environment”). For each item, participants are instructed to rate their responses on a Likert scale ranging from 1 (“completely disagree”) to 7 (“completely agree”). The internal consistency of the global score was good with *α* = 0.91 (*M* = 5.10, SD = 0.77). We used the TEIQue-SF for validity analyses because interoception showed meaningful relationships with emotion regulation (Lischke et al., 2020).

#### Giessener complaint questionnaire

Psychosomatic symptoms were found to be related with interoceptive sensibility (Ciaramella et al., 2023). Therefore, we assessed psychosomatic contributions to physical symptoms with the Giessen Complaint Questionnaire (GBB-24; Brähler et al., 2008). The GBB-24 assesses physical complaints in the areas of (a) exhaustion, (b) stomach/gut complaints, (c) limb pain, and (d) heart complaints. The overall score allowed us to assess the burden of complaints with a mean score of *M* = 1.70 (SD = 0.47; *α* = 0.88).

#### Toronto alexithymia scale

The Toronto Alexithymia Scale (TAS-20; Bagby et al., 1994) is a widely used instrument in research for assessing alexithymia (Bagby et al., 2020). The questionnaire consists of 20 statements rated on a five-point Likert scale ranging from “does not apply at all” to “fully applies”. We used the German version (Popp et al., 2008), which demonstrated good reliability of α = 0.85 for the total score (*M* = 2.18, SD = 0.55). Previous research indicated weak but significant relationships with interoceptive sensibility (Zamariola et al., 2018).

#### Perceived stress scale

Because research indicated associations between stress and interoception (Shen et al., 2023; Schulz and Vögele, 2015), we used the perceived stress scale (PSS; Cohen et al., 1983) to quantify the subjective perception of recent stressful life circumstances (see also Harris et al., 2023). We used the German version with 10 items, which were rated on a 5-point Likert scale from 1 (“never”) to 5 (“very often”; Schneider et al., 2020). The internal consistency was α = 0.89, with *M* = 2.80, SD = 0.78.

#### Physical activity

Physical activity was assessed because it was shown to be associated with interoceptive sensibility (e.g., Georgiou et al., 2015). We used 3 items which are based on the physical activity recommendations of the WHO (e.g., “I perform muscle-strengthening activities of moderate or high intensity that involve all muscle groups on 2 or more days per week”). Participants responded on a 5-point Likert scale. For the analysis, a mean score from the three items was calculated with *M* = 2.86 (SD = 1.01, *α* = 0.73).

#### Psychometric data analytic strategy

We calculated reliability analyses on the between- (*R_KR_*) and the within-person (*R_cn_*) level using generalizability theory analysis (GTA; Brennan, 2001; Cronbach, 1972, for a review see, Shrout and Lane, 2012). We differentiated between Level 1, which were the three items for interoceptive intensity and for interoceptive valence, Level 2, which were the prompts, and Level 3 as the person level (Nezlek, 2017). GTA is especially suited to assess between-person and within-person reliability of EMA data by decomposing the observed variance associated with person, item, prompt, and their interactions. To estimate reliability, we used the methods described by Revelle and Wilt (2019) by applying the software psych (version 2.4.6.26; Revelle, 2023) running in R (version 4.4.1; R Core Team, 2023) and utilizing lme4 package (Bates et al., 2015). Crucially, because this estimation relies on Linear Mixed-Effects Models, it natively handles unequal numbers of observations per participant due to missed prompts by using all available data. Secondly, to estimate the stability of the measure, the scores based on the first half of prompts were correlated with the scores based on the second half of prompts.

For validity estimates at the between-person level, aggregated scores for interoceptive intensity and valence, respectively, were correlated with relevant trait questionnaires (e.g., PSS; GBB, SHS). The correlations evaluating the relationships between MIIS and MIVS and the validation measures constitute our primary construct validation analyses. The remaining intercorrelations among the external psychological and demographic covariates are provided for complete transparency but are explicitly designated as exploratory background data. To evaluate the within-person validity, we calculated one multilevel model predicting the score of the MIIS with the MIVS rating at Level 1 and Level 2 as fixed effects. We additionally included a quadratic term to evaluate potentially curvilinear relationships. Furthermore, we calculated two separate multi-level models with participants as random effects predicting the MIIS and MIVS, respectively, for each prompt via pleasure, arousal, dominance, hunger, and pain at Level 1 (within) and Level 2 (between) as fixed effects. All Level 1 variables (i.e., within-person) were group mean centered. As Level 2 variables (i.e., between-person variation) served the means of the variables per person. We additionally added the location (at home vs. elsewhere) and social contact (alone vs. in contact) as factors. For all multilevel model analyses random intercepts for participants, as well as random slopes for Level 1 within-person predictors were entered to account for individual differences in reactivity. If the models with random slopes failed to converge, we stayed with random intercept only models (Matuschek et al., 2017). Furthermore, to explicitly model the residual error structure, we applied a continuous-time first-order autoregressive correlation structure (corCAR1) using the nlme package (version 3.1–164, Pinheiro et al., 2023). Assumptions of normality and homoscedasticity were adequately met (QQ-plots and residual-versus-fitted plots).

## Results

### Descriptive statistics

Overall, 5,913 EMA responses were available for analysis. On average, participants provided 41.94 (49.93% of all planned prompts; *SD* = 17.85) prompts. The minimum number of valid prompts (i.e., all questions were answered) per participant varied between 11 (13.09%) and 80 (95.24%).

### Reliability of the MIIS and MIVS

The between-person reliability was high for both measures ( $R_{\mathrm{KRn}}$ = 0.99). The within-person reliability was sufficient with $R_{\mathrm{Cn}}$ = 0.60 and 0.42, although at the lower boundary for the MIIS (see Table 1). This indicates that differences between prompts reliably reflected systematic within-person variations of both indices. Importantly, 25–36% of variance was due to person and 17–22% due to the interaction of person and time, thus indicating sufficient within-person variance for multilevel modeling. Nonetheless, the analysis revealed that a considerable proportion of variability stemmed from the interaction between individuals and specific items, amounting to 11% for intensity scores and 7% for valence scores.

**Table 1:** Variance decomposition of interoceptive intensity (MIIS) and interoceptive valence (MIVS) and summary of reliability estimates.

	MIIS		MIVS	
Variance component				
$\sigma_{\text{Total}}^{2}$	520.10		470.65	
$\sigma_{P}^{2}$	124.97	24%	170.70	36%
$\sigma_{T}^{2}$	4.95	1%	0.00	0%
$\sigma_{I}^{2}$	15.22	3%	9.43	2%
$\sigma_{P*T}^{2}$	88.92	17%	104.63	22%
$\sigma_{P*I}^{2}$	59.92	11%	29.06	7%
$\sigma_{T*I}^{2}$	2.75	1%	1.01	0%
$\sigma_{\text{Residuals}}^{2}$	223.36	43%	155.83	33%
GT reliability estimates				
$R_{\mathrm{KRn}}$	0.99		0.99	
$R_{\mathrm{Cn}}$	0.42		0.60	

$\sigma^{2}$ = variance component, P = person, T = time, I = item; $R_{\mathrm{KRn}}$ = nested between-person reliability; $R_{\mathrm{Cn}}$ = nested within-person reliability. MIIS, Momentary interoceptive intensity scale. MIVS, Momentary interoceptive valence scale.

### Stability of the measures to assess between-person parameters

To explore the stability of the scores, we analyzed if between-person estimates of the first half of prompts were correlated with the estimates of the second half of prompts. This analysis showed evidence of good stability of both, the MIIS (interoceptive intensity; *r* = 0.83) and the MIVS (valence; *r* = 0.87; see Figure 1).

**Figure 1:**
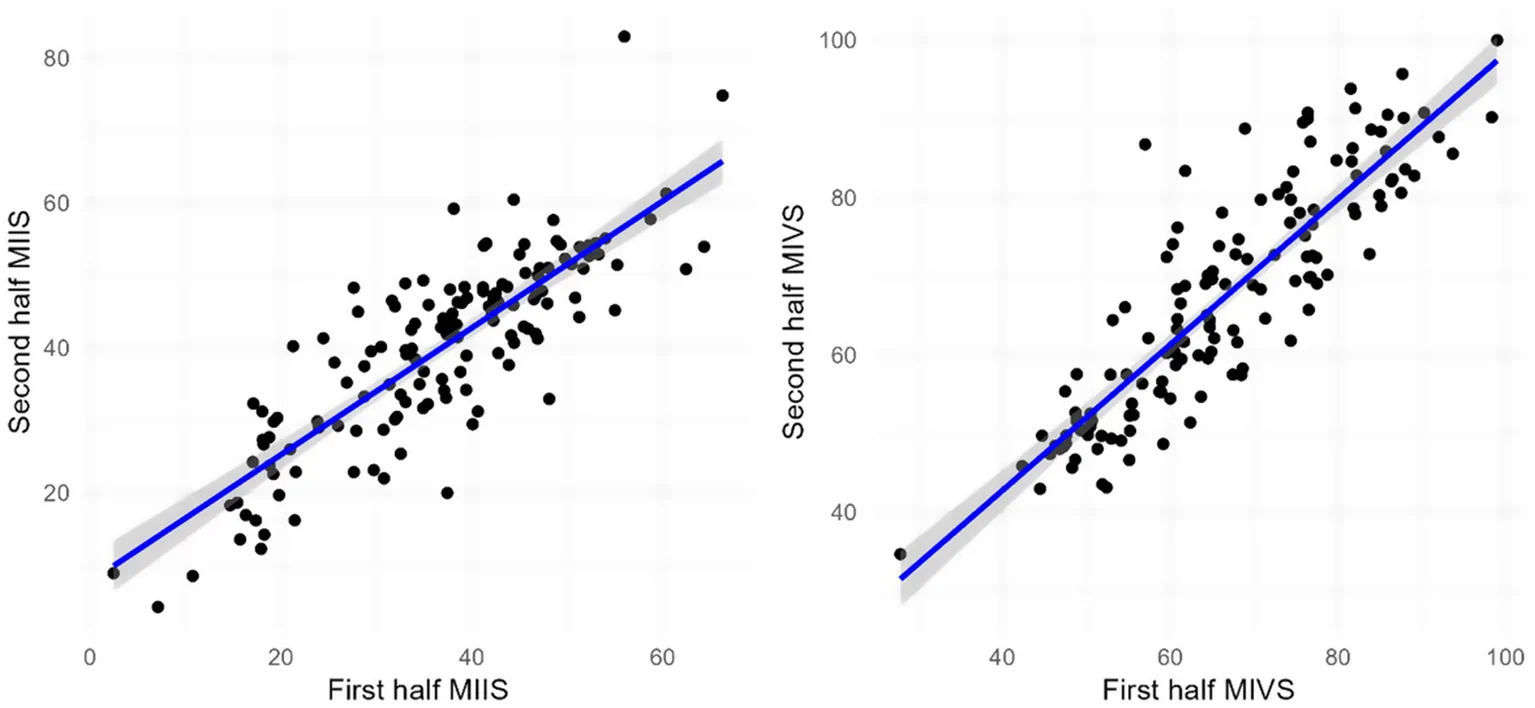
Split-half stability of MIIS (interoceptive intensity, left) and MIVS (valence ratings, right). Slope and corresponding credibility intervals are illustrated.

### Validity analysis

#### Between-person validity of the MIIS and the MIVS

As illustrated in Table 2, the primary analyses indicated that MIIS was significantly and negatively associated with the MIVS scores (*r* = −0.52, *p* < 0.001) at the between-person level, indicating that both variables share meaningful variance. People who perceived their bodily signals more strongly also perceived their bodily signals as less positive. Furthermore, the MIIS score was not significantly associated with any further between-person variable. However, the MIVS score was associated with most variables of interest, except physical activity, age, and gender. Specifically, people who indicated more pleasant interoceptive sensations also scored higher on questionnaires such as the BAS-2 (Body Appreciation Scale), SHS (Subjective Happiness Scale), FFMQ (Five Facet Mindfulness Scale), IES-2 (Intuitive eating scale). Furthermore, interoceptive valence was negatively associated with the TAS-20 (mean score, Toronto Alexithymia scale), the PSS (Perceived Stress Scale), and the GBB (Giessener Complaint Questionnaire), providing evidence for the validity of the MIVS score at Level 2.

**Table 2:** Pearson correlations between interoceptive intensity (MIIS) and interoceptive valence (MIVS) at the between-person Level 2.

Variable	1. MIIS	2. MIVS	3. Gender	4. Age	TAS-20	PSS	GBB	TEIQue	BAS-2	FFMQ	IES-2	SHS	Sport
1. MIIS	–	[−0.63, −0.39]	[−0.16, 0.18]	[−0.15, 0.18]	[−0.07, 0.25]	[−0.08, 0.25]	[−0.03, 0.30]	[−0.25, 0.08]	[−0.19, 0.14]	[−0.21, 0.12]	[−0.21, 0.12]	[−0.25, 0.08]	[−0.14, 0.19]
2. MIVS	−0.52 (< 0.001)	–	[−0.22, 0.11]	[−0.20, 0.13]	[−0.37, −0.06]	[−0.50, −0.21]	[−0.50, −0.22]	[0.23, 0.51]	[0.12, 0.43]	[0.21, 0.50]	[0.11, 0.42]	[0.20, 0.49]	[−0.07, 0.26]
3. Gender	0.01 (0.903)	−0.05 (0.529)	–	[0.08, 0.39]	[−0.24, 0.09]	[−0.31, 0.01]	[−0.39, −0.07]	[−0.13, 0.21]	[−0.10, 0.23]	[−0.05, 0.28]	[−0.13, 0.20]	[−0.21, 0.12]	[−0.17, 0.16]
4. Age	0.01 (0.894)	−0.04 (0.657)	0.24 (0.005)	–	[−0.21, 0.12]	[−0.22, 0.11]	[−0.22, 0.11]	[−0.13, 0.20]	[−0.11, 0.22]	[−0.12, 0.21]	[−0.22, 0.11]	[−0.14, 0.19]	[−0.16, 0.17]
5. TAS-20	0.09 (0.277)	−0.22 (0.009)	−0.08 (0.350)	−0.05 (0.567)	–	[0.36, 0.61]	[0.25, 0.53]	[−0.77, −0.59]	[−0.59, −0.33]	[−0.72, −0.52]	[−0.36, −0.05]	[−0.58, −0.32]	[−0.26, 0.07]
6. PSS	0.09 (0.313)	−0.36 (< 0.001)	−0.15 (0.074)	−0.06 (0.500)	0.50 (< 0.001)	–	[0.29, 0.56]	[−0.72, −0.52]	[−0.67, −0.45]	[−0.68, −0.46]	[−0.40, −0.09]	[−0.64, −0.41]	[−0.35, −0.03]
7. GBB	0.14 (0.097)	−0.37 (< 0.001)	−0.23 (0.006)	−0.06 (0.497)	0.40 (< 0.001)	0.44 (< 0.001)	–	[−0.51, −0.23]	[−0.55, −0.28]	[−0.58, −0.31]	[−0.41, −0.10]	[−0.42, −0.12]	[−0.29, 0.04]
8. TEIQue-SF	−0.09 (0.301)	0.38 (< 0.001)	0.04 (0.626)	0.04 (0.635)	−0.69 (< 0.001)	−0.63 (< 0.001)	−0.38 (< 0.001)	–	[0.54, 0.73]	[0.69, 0.83]	[0.17, 0.47]	[0.60, 0.77]	[0.02, 0.34]
9. BAS-2	−0.02 (0.802)	0.28 (< 0.001)	0.07 (0.439)	0.06 (0.489)	−0.47 (< 0.001)	−0.57 (< 0.001)	−0.42 (< 0.001)	0.65 (< 0.001)	–	[0.48, 0.69]	[0.41, 0.64]	[0.47, 0.69]	[0.01, 0.33]
10. FFMQ	−0.05 (0.560)	0.37 (< 0.001)	0.12 (0.156)	0.04 (0.597)	−0.63 (< 0.001)	−0.58 (< 0.001)	−0.45 (< 0.001)	0.77 (< 0.001)	0.60 (< 0.001)	–	[0.16, 0.45]	[0.41, 0.65]	[0.04, 0.35]
11. IES-2	−0.05 (0.585)	0.27 (0.001)	0.04 (0.649)	−0.06 (0.493)	−0.21 (0.013)	−0.25 (0.003)	−0.26 (0.002)	0.33 (< 0.001)	0.54 (< 0.001)	0.31 (< 0.001)	–	[0.22, 0.51]	[−0.15, 0.18]
12. SHS	−0.09 (0.289)	0.35 (< 0.001)	−0.04 (0.602)	0.02 (0.773)	−0.46 (< 0.001)	−0.54 (< 0.001)	−0.28 (< 0.001)	0.70 (< 0.001)	0.59 (< 0.001)	0.54 (< 0.001)	0.37 (< 0.001)	–	[−0.02, 0.30]
13. Sport	0.03 (0.737)	0.10 (0.245)	−0.00 (0.955)	0.01 (0.938)	−0.10 (0.242)	−0.19 (0.021)	−0.13 (0.137)	0.19 (0.025)	0.18 (0.034)	0.20 (0.017)	0.02 (0.827)	0.14 (0.089)	–

MIIS (Momentary interoceptive intensity scale), MIVS (Momentary interoceptive valence scale), BAS-2 (Body Appreciation Scale), SHS (Subjective Happiness Scale), FFMQ (Five Facet Mindfulness Scale), IES-2 (Intuitive eating scale), TAS-20 (Toronto Alexithymia scale), PSS (Perceived Stress Scale), GBB (Giessener Complaint Questionnaire). Exact *p*-values are in parentheses, and 95% confidence intervals are reported for all correlations in the upper diagonal.

#### Within-person validity

##### Association between momentary interoceptive intensity and interoceptive valence

As shown in Table 3, higher interoceptive intensity ratings were associated with less pleasant interoceptive sensations at Level 2 as well as Level 1 explaining 21% of the total variance. When adding quadratic terms, we found a significant negative quadratic effect of situational valence, indicating a curvilinear relationship where the negative association between valence and intensity is stronger at higher levels of valence (see Supplementary Table 1).

**Table 3:** Interoceptive intensity (MIIS) predicted via interoceptive valence (MIVS) at Level 1 and Level 2.

Predictors	Estimates	Conf. Int (95%)	*p*-value
Intercept	68.35	59.76–76.93	<0.001
MIVS trait (Level 2)	−0.45	−0.58 – −0.33	<0.001
MIVS state (Level 1)	−0.44	−0.49 – −0.39	<0.001
Random effects			
σ^2^	134.41		
τ_00 Participant_	104.29		
τ_11 Participant. MIVS (Level 1)_	0.07		
ρ_01 Participant_	0.20		
ICC	0.46		
N _Participant_	141		
Observations	5,907		
Marginal *R*^2^/Conditional *R*^2^	0.211/0.574		

MIVS, Momentary interoceptive valence scale.

##### Associations of MIIS and MIVS with momentary affect, hunger, and pain

Pleasure ratings were associated with both variables at Level 1 and with the MIVS at Level 2, indicating more positive interoceptive valence and less intense bodily sensations in cases of higher feelings of pleasure. Furthermore, hunger and pain were linked with both interoceptive intensity and valence, with greater hunger being associated with lower valence and higher intensity of interoceptive sensations. The same applied for pain, with higher pain ratings being associated with more intense interoceptive sensations and lower valence of those sensations. Interestingly, the presence of other people did not show strong associations with both interoceptive measures. Being at home (vs. not), in turn, was associated with slightly more pleasant interoceptive sensations (at a trend; see Table 4).

**Table 4:** Multilevel model predicting interoceptive intensity and valence with relevant variables at Level 1 and Level 2.

Predictors	Interoceptive intensity (MIIS)			Interoceptive valence (MIVS)		
	Estimates	Conf. Int (95%)	*p*-value	Estimates	Conf. Int (95%)	*p*-value
Intercept	24.25	4.96–43.55	0.014	53.79	33.51–74.07	<0.001
Pleasure (Level 2)	−1.42	−5.25 – 2.42	0.466	6.74	2.71–10.77	0.001
Pleasure (Level 1)	−0.97	−1.41 – −0.53	<0.001	2.47	2.06–2.89	<0.001
Arousal (Level 2)	4.94	1.78–8.10	0.002	−5.30	−8.63 – −1.97	0.002
Arousal (Level 1)	2.93	2.55–3.31	<0.001	−2.60	−2.96 – −2.23	<0.001
Dominance (Level 2)	−0.41	−3.41 – 2.59	0.788	3.10	−0.05 – 6.25	0.053
Dominance (Level 1)	0.99	0.53–1.45	<0.001	0.56	0.12–1.00	0.012
Hunger (Level 2)	0.38	0.22–0.55	<0.001	−0.26	−0.43 – −0.09	0.003
Hunger (Level 1)	0.04	0.03–0.06	<0.001	−0.02	−0.03 – −0.01	0.003
Pain (Level 2)	−0.11	−0.30 – 0.07	0.232	−0.12	−0.32 – 0.08	0.241
Pain (Level 1)	0.12	0.08–0.15	<0.001	−0.17	−0.20 – −0.14	<0.001
Location (at home)	−0.62	−1.43 – 0.19	0.132	0.73	−0.00 – 1.46	0.051
Alone vs. in contact	0.05	−0.73 – 0.83	0.907	0.44	−0.29 – 1.17	0.239
Random effects						
σ^2^	162.11			143.13		
τ_00_	114.71 _Participant_			129.57 _Participant_		
ICC	0.41			0.48		
N	141 _Participant_			141 _Participant_		
Observations	5,905			5,905		
Marginal *R*^2^/Conditional *R*^2^	0.128/0.489			0.190/0.575		

Because the within-person reliability of the MIIS was at the lower bound with 0.42, we additionally fitted the multilevel models separately for each of the three MIIS items (see Supplementary Table 4). Overall, the results showed a relatively homogeneous pattern, with most effects in the same direction, though not always statistically significant for all items. In contrast, the two context variables location and social contact showed markedly different effects for breath versus gut, suggesting that the observed effects were context dependent to some degree.

### Discussion study 1

We conducted study 1 to initially explore the psychometric properties of the MIIS and MIVS, assessing interoceptive attention and appraisal of body sensations in everyday life situations. This EMA approach aimed to address the limitations and problems of more classic laboratory approaches and questionnaires, such as questionable ecological validity, lack of differentiation between within- and between-person variation in interoception, unknown generalization of findings across interoceptive domains, and retrospective bias (Poerio et al., 2024). In case of promising findings, the presented method for ambulatory interoception could scale up, because researchers could apply this method fully remote, which allows the recruitment of large and diverse samples.

Importantly, findings indicated that both interoceptive intensity and valence as indices of attention and appraisal can be measured reliably at the within- and between-person levels in ambulatory settings, showing good stability at Level 2 across 1 week. Thus, subjective interoceptive intensity and valence can be assessed reliably across different individuals. Across different domains and situations, the reliability of the MIIS seems only modest with 0.42. Nezlek (2017) discussed that within-person reliabilities are systematically lower than between-person reliabilities and, in EMA measures, within-person reliabilities in the 0.40 range can be considered sufficient to capture momentary fluctuations. Nevertheless, interpretations of within-person changes should take the lower reliability of the MIIS into account. Further sensitivity analyses, however, confirmed quite homogeneous associations across the interoceptive domains. But what does this study tell us about the validity of these two novel ambulatory scales?

First, we should differentiate within-person and between-person evidence for validity. The analysis inspecting the between-person validity showed that ambulatory assessed interoceptive intensity (MIIS) was not strongly associated with any trait variable included in this study. Hence, individuals with higher attention to sensations from the body in everyday life could not be characterized by any of the trait measures applied. However, within-person fluctuation of intensity matters (see below), which underlines its scientific value. In contrast, interoceptive valence (MIVS) was robustly associated with most of the between-person variables, indicating evidence for convergent validity of the MIVS. In line with interoception literature, which has documented robust associations between interoceptive measures and a broad range of positive and negative health-relevant traits, the overall MIVS score (mean per person) was significantly positively associated with questionnaires assessing happiness (Ferentzi et al., 2019), intuitive eating (Poovey et al., 2022), mindfulness (Hanley et al., 2017), body appreciation (Todd et al., 2020), and negatively with questionnaires assessing physical complaints, alexithymia (Zamariola et al., 2018), and perceived stress (Schulz and Vögele, 2015). These findings highlight the importance of ambulatory interoceptive valence as a measure of interoceptive appraisal for interoception research targeting health and well-being. However, it should be kept in mind that the MIVS score was significantly and negatively associated with the mean score of the MIIS at both Level 2 and Level 1, which showed similar (although non-significant) patterns of associations with the validity indices in study 1. This indicates the complementary relevance of the MIIS for studying interoception, where the MIIS captures how intense the body signal is felt and the MIVS captures how it is evaluated (pleasant vs. unpleasant). However, future research directly comparing these pathways is needed to formally determine if valence is a consistently stronger driver of well-being than intensity.

Second, at the within-person level, we found a comparable pattern of results for both interoceptive intensity ratings and valence, again in different directions. Momentary valence and intensity showed a negative association at Level 1 (similar to Level 2). Furthermore, situations with more negative sensations such as hunger and pain were associated with higher attention (i.e., perceived intensity) and lower valence (Vaitl, 1996; Craig, 2002). Intense bodily sensations can often reflect symptoms, discomfort, or stress (e.g., stronger heart pounding, discomfort in the gut, breathing difficulty), which would naturally co-occur with lower valence and poor well-being and pleasure. At the within-person level, higher interoceptive valence was associated with more pleasure and lower arousal, whereas higher interoceptive intensity showed the opposite pattern (less pleasure, higher arousal), which further strengthens this interpretation and the validity of both scales. Although aspects of interoception (such as interoceptive accuracy) are often positively linked with self-regulation and emotional control (Lischke et al., 2020; Schultchen et al., 2019; Rominger and Schwerdtfeger, 2023), the current results argue that ambulatory assessed interoceptive intensity as an indicator of interoceptive attention is more likely negatively linked with interoceptive valence and affect in everyday life. The divergent relationship of both measures with pain and hunger again underscores the complementary value of both dimensions to understand the role of bodily sensations in everyday life.

Taken together, study 1 indicated that we can assess interoceptive intensity and valence moderately reliably in diverse daily life contexts. We additionally found evidence for the validity of both measures at the person and the state level. The divergent pattern of findings for valence and intensity ratings also indicated some independence of both measures, thus arguing for two distinct but related and equally important dimensions of interoception in everyday life settings. This study adds a reliable instrument to complement the assessment of ambulatory interoception with a measure of the hedonic component of interoception (i.e., appraisal; in terms of valence in everyday life contexts) and intensity (i.e., attention) in literature. Of note, although previous theoretical work advocated for the role of valence and appraisal (see, e.g., Khalsa et al., 2018; Feldman et al., 2024), to the best of our knowledge, no such instrument has been introduced to date (see, e.g., Poerio et al., 2024 for a more direct ambulatory assessment of interoceptive attention).

We found that interoceptive valence was negatively associated with the perceived intensity of bodily cues, indicating that the linear relationship got more negative at higher levels of valence. Furthermore, perceived intensity was less consistently linked to other relevant variables, which suggests that the primary focus on the intensity of interoception might fall short for some specific study questions. This is in accordance with Poerio et al. (2024), who concluded from their results that high ratings of within-person interoceptive attention may not be considered a positive, adaptive characteristic, our results suggest that stronger bodily sensations are not necessarily beneficial either. Therefore, it may be important in terms of health and well-being how positive/negative participants perceive their bodily signals and not that much how intense. The valence measure (MIVS) exactly displayed the expected pattern of findings, documenting that higher well-being seems to be associated with more positive perceptions of bodily signals. In contrast, stronger or more intense interoceptive sensations might be associated with less positive feelings (Poerio et al., 2024; MacVittie et al., 2025). Experiencing the body in a positive way demonstrates the expected beneficial aspects of interoception in everyday life contexts. To conclude, our initial findings confirm that interoceptive experiences in everyday life are important and can be captured as both stable between-person differences and systematic within-person fluctuations (Poerio et al., 2024), and including interoceptive valence broadens the perspective on interoceptive experience in daily life. Notably, stronger bodily sensations in daily life are more often unpleasant than pleasant.

### Study 2

In light of the promising findings of study 1, we conducted study 2 to add further evidence for the usefulness of a two-dimensional perspective on ambulatory interoception. Study 1 did not directly investigate associations with questionnaires specifically designed to assess subjective interoceptive awareness (i.e., MAIA-2; Mehling et al., 2018). Furthermore, study 1 did not explicitly ask for body awareness on a momentary basis in everyday life (MacVittie et al., 2025; MacVittie et al., 2024) to provide further support for within-person validity. In order to replicate the promising reliability estimates in study 1 and to advance evidence for convergent validity, study 2 aimed to add a classic interoceptive sensibility questionnaire (MAIA-2; Mehling et al., 2018) as well as further psychologically meaningful variables presumably associated with interoception. To further explore validity of our ambulatory measurements we collected data on interoception-relevant psychological constructs such as anxiety sensitivity, resilience, depression, perceived safety, and high sensitivity (see, e.g., Solano López and Moore, 2019; Domschke et al., 2010; Ujiie and Takahashi, 2024). At the within-person level, in addition to affect, we collected information on psychological safety and exteroception, variables potentially relevant for interoception (Nord and Garfinkel, 2022). In sum, study 2 aimed to replicate and extend the psychometric findings of study 1 regarding the ambulatory assessment of MIIS and MIVS in order to arrive at robust and valid ambulatory measures of subjective interoception, covering both intensity and valence as measures of interoceptive attention and appraisal (Khalsa et al., 2018; Murphy et al., 2020; Farb et al., 2015).

### Methods of study 2

#### Participants

In study 2, a total of 132 participants took part. We excluded eight participants because they only answered one prompt. Three participants did not provide information on the between-person variables. One participant was excluded because of extremely low ratings on the MIVS with more than 3.5 SDs below the mean. While this extreme score may not reflect invalid responses, we excluded this participant to preserve the integrity of the sample-level correlations. Furthermore, as for study 1, we excluded prompts with a duration shorter than 0.5 min and longer than 10 min. After this, only participants with more than 10 valid answered prompts were included in the main analyses. The final sample consisted of 112 participants with 5,503 valid prompts. In line with study 1, study 2 had a sufficient power (≥0.80) to detect small within-person effects with a significance level of 0.05 (Arend and Schäfer, 2019). The mean age of the participants (92 women, 17 men, 3 diverse) was 25.45 years (SD = 9.96). All participants gave written informed consent prior to the assessment days in the Esmira app running on a local server of the university (Lewetz and Stieger, 2023). Participants received course credits for participation. The ethical committee of the University of Graz approved study 1 and study 2 (GZ. 39/82/63 ex 2014/15).

#### Procedure and study design

Similar to the procedure of study 1, the EMA took place for 8 days, starting at a random day. The Esmira app (Lewetz and Stieger, 2023) delivered 10 random prompts per day (08:00 a.m. - 08:00 p.m.). The minimum distance between random prompts was defined by 30 min. Participants could delay the prompts if they wished for five times with 25 min in total. There were no time limits when answering a prompt. At the beginning of the assessment participants got written instructions and were requested to fill out questionnaires. The study was conducted from 25.11.2024 to 03.02.2025.

#### Within-person variables

##### MIIS

As in study 1, we used the very same items of the momentary interoceptive intensity scale (MIIS; Walentynowicz et al., 2023). Participants rated their interoceptive sensations using a visual analog scale ranging from 1 to 100. A score of 1 represents minimum intensity, while a score of 100 represents maximum intensity. Comparable, though somewhat lower than in study 1, the mean of all three items (heart, breath, gut) served as total score for interoceptive intensity (*M* = 34.70, SD = 17.12).

##### MIVS

As in study 1, we used the very same items of the MIVS. Participants rated the items on a scale from 1 (unpleasant) to 100 (pleasant). We measured interoceptive valence of sensation of the heart (at this moment, my heartbeat feels), breath (at this moment, my breathing feels), and stomach/gut (at this moment, my gut feels). We used the mean over all three items per prompt as a measure of interoceptive valence. The mean of 68.72 (SD = 17.60) was quite comparable to study 1.

##### Momentary affect

As in study 1, we assessed current affective states with the SAM (Bradley and Lang, 1994) assessing pleasure, arousal, and dominance. The mean score for pleasure was 3.87 (SD = 0.89), for arousal was 2.29 (SD = 1.01), and for dominance was 2.37 (SD = 0.99; with higher scores indicating higher dominance).

##### Momentary body awareness

To assess momentary variability of body awareness we used the single item “How aware are you of any sensations from your body (e.g., your heartbeat, your breathing, your stomach/gut, your skin or muscles)?” (MacVittie et al., 2025). Participants rated their in-the moment body awareness on a 7-point scale (from 1 = not at all aware to 7 = completely aware): This item was used in this study to capture overall awareness of any sensations from the body regardless of intensity or quality. The mean score in this study across all prompts and participants was *M* = 4.20, (SD = 1.75).

##### Perceived safety

The perception of safety in everyday life was assessed using a single item. Participants were asked to rate, on a five-point Likert scale, how safe they felt at the given moment (see, e.g., Schwerdtfeger et al., 2022; Schwerdtfeger and Rominger, 2024b). The scale ranged from one to five, with higher scores indicating a greater sense of safety (*M* = 4.40, SD = 0.77). Of note, feeling more safe was found to be correlated with physiological states of reduced activation in terms of lower heart rate and higher heart rate variability in prior research (Schwerdtfeger et al., 2022), which might be linked with interoceptive valence or intensity.

##### Loudness and general intensity of surroundings

We used a self-generated single-item measure to assess the subjectively perceived environmental noise level. The item reads: “How loud do you currently perceive your environment to be?” and could be answered using a visual analog scale ranging from “quiet” (0) to “loud” (100). The mean score was *M* = 31.55 (SD = 21.97). We assessed this because exteroception is an important complement to interoception (Nord and Garfinkel, 2022).

##### Additional contextual information

At each prompt (*n* = 5,503), participants indicated whether they were in contact with other people (2,008 prompts) vs. being alone (3,495 prompts). Participants further indicated their current location, that is being at home (3,469 prompts) or being somewhere else (2,034 prompts).

#### Between-person variables

##### MAIA-2

To assess interoceptive sensibility, we used the German version of the Multidimensional Assessment of Interoceptive Awareness (MAIA-2; Mehling et al., 2018), which is widely used in interoception research (Desmedt et al., 2025; Vig et al., 2022). The MAIA-2 is a questionnaire originally developed and validated in English, which measures interoception across eight subscales: Noticing, Not-Distracting, Not-Worrying, Attention Regulation, Emotional Awareness, Self-Regulation, Body Listening, and Trust. The items of the MAIA-2 were answered on a six-point Likert scale, with higher values indicating greater interoceptive sensibility. The internal consistency for the eight subscales ranged between α = 0.64 and α = 0.87. We also calculated a sum score with *α* = 0.88 (*M* = 3.23, SD = 0.53).

##### STADI

Depressive symptoms have been associated with compromised interoception (Solano López and Moore, 2019). Therefore we measured depressive tendencies via 10 items addressing trait depression from the German State–Trait Anxiety-Depression Inventory (STADI; Laux et al., 2013). Participants were instructed to indicate how they generally characterize themselves, as depression in this case was assessed as a trait of the person. The questions focused on the frequency of experienced feelings and were answered using a four-point Likert scale, with 1 (“rarely”), 2 (“sometimes”), 3 (“often”), and 4 (“almost always”) as response options. Examples of items include: “I feel hopeless.” and “I feel empty.” Half of the items (assessing euthymic traits) were reverse-coded, such as “I enjoy life.” and “I feel cheerful.” The internal consistency of the trait depression was α = 0.91. The mean score was 1.97 (SD = 0.55).

##### CD-RISC

The ability to perceive interoceptive signals might be associated with resilience and well-being (May and Tapert, 2025). To evaluate this further, resilience was measured using the German version of the Connor-Davidson Resilience Scale (CD-RISC; Connor and Davidson, 2003; Sarubin et al., 2015). The original scale identifies five factors: (I) Acceptance of change and secure relationships, (II) Trust in instincts, tolerance of negative affect, and strengthening effects of stress, (III) Personal competence, high standards, and perseverance, (IV) Control, and (V) Faith. Since the factor structure could not be replicated for the German version, the corresponding subscales were not interpreted in this study. The German version of the CD-RISC consists of 25 items. Participants indicated their agreement with statements describing their resilience over the past month on a five-point Likert scale, ranging from “not true at all” to “almost always true.” The items were presented in randomized order to avoid order effects. The internal consistency was good with 0.90. The mean score was 3.66 (SD = 0.53).

##### HSPS

The Highly Sensitive Person Scale (HSPS) by Aron and Aron (1997) was used to assess individual levels of sensitivity, because previous research indicated a positive link with interoceptive sensitivity (Ujiie and Takahashi, 2024). While the original version consists of 27 items, this study utilized the 25 items short version by Smolewska et al. (2006). Items can be divided into three subscales: Ease of Excitation (EOE), Low Sensory Threshold (LST), and Aesthetic Sensitivity (AE). Examples of items include: “Do you seem to notice subtle things in your surroundings?” and “Do you react particularly sensitively to coffee?” The items are rated on a Likert scale from one (“strongly disagree”) to seven (“strongly agree”; Smolewska et al., 2006). The resulting scale demonstrated high internal consistency (*α* = 0.84) with a mean score of 4.94 (SD = 0.72).

##### MDDI

We used the German Version of the MDDI (Muscle Dysmorphic Disorder Inventory; Zeeck et al., 2018; Hildebrandt et al., 2004) to assess symptoms of muscle dysmorphia. Previous research indicated a link with interoception (Grunewald et al., 2023). It consists of 13 items, divided into three subscales. The items use a five-point Likert scale, ranging from 1 (“never”) to 5 (“always”). The overall questionnaire score is calculated by summing up the subscales. The three subscales are Drive for Size (DS): Captures the desire to increase muscle mass, along with related thoughts and behaviors (*α* = 0.81). Appearance Intolerance (AI): Measures dissatisfaction with one’s appearance and intolerance toward one’s body image (*α* = 0.81). Functional Impairment (FI): Assesses the restrictions and impairments that affect behavior and well-being in connection with muscle dysmorphia (*α* = 0.80). The internal consistency of the overall score was 0.75. with mean of 2.11 (SD = 0.58).

##### NPSS-G

The general perception of psychological safety was assessed using the German version of the Neuroception Psychological Safety Scale (NPSS-G; Morton et al., 2024; Schneider et al., 2026). We used this scale because previous research indicated associations with physiological states such as reduced heart rate and increased heart rate variability, which might be associated with interoceptive sensations (Schwerdtfeger et al., 2025). The NPSS-G consists of 29 items divided into three subscales: Social Engagement (*α* = 0.93), Compassion (*α* = 0.88), and Body Sensations (*α* = 0.91). The items are rated on a five-point Likert scale, where higher scores indicate a stronger sense of psychological safety. The questionnaire demonstrated high internal consistency of *α* = 0.94 with a mean score of 3.96 (SD = 0.58).

##### ASI-3

Because anxiety sensitivity has been associated with interoception (Domschke et al., 2010), we used the German version of the Anxiety Sensitivity Index-3 (ASI-3; Kemper et al., 2009). The questionnaire consists of 18 items, distributed across three subscales: social concerns (*α* = 0.76), cognitive concerns (*α* = 0.83), and somatic concerns (*α* = 0.82). Participants provided responses on a five-point Likert scale, ranging from 0 (strongly disagree) to 4 (strongly agree). The internal consistency of the ASI-3 was good with Cronbach’s α of 0.88 and a mean score of 2.71 (SD = 0.71).

##### Psychometric data analytic strategy

Similar to study 1, we first estimated reliability applying GTA (Brennan, 2001). Then, we calculated the stability of the between-person estimates of the MIIS and MIVS, respectively. To indicate the between-person validity indicators, we calculated correlations between the mean scores across all prompts per person and the between-person questionnaires (e.g., MAIA, CD-RISC). Finally, we calculated multilevel models with relevant variables at both the between- and within-person level, to predict the situational variation of the MIIS and the MIVS. We applied the same statistical methods as described for study 1. All statistical analyses were at the significance level of *p* < 0.05.

### Results of study 2

#### Descriptive statistics

Overall, 5,503 EMA prompts were available for analysis. Participants provided 49.13 (61% of planned prompts; SD = 19.77) prompts on average. The minimum number of valid prompts per participant was 11 (13.75%) and the maximum was 79 (98.75%).

#### Reliability for interoceptive intensity and interoceptive valence

The between-person reliability was high for both measures ( $R_{\mathrm{KRn}}$ = 0.99 and 0.98). The within-person reliability was sufficient with $R_{\mathrm{Cn}}$ = 0.67 and 0.52 (see Table 5). This indicates that differences between prompts reliably reflect systematic within-person variations for both indices. Importantly, 24–33% of the variance was due to person and 24–29% was due to the interaction of person and time, thus indicating sufficient within-person variance for multilevel modeling. Interestingly, the interaction between person and item was again 12 and 9%, respectively, indicating that people differentially answered the three items per scale.

**Table 5:** Replication of variance decomposition of interoceptive intensity (MIIS) and interoceptive valence (MIVS) and summary of reliability estimates.

	MIIS		MIVS	
Variance component				
$\sigma_{\text{Total}}^{2}$	447.21		409.60	
$\sigma_{P}^{2}$	108.69	24%	134.76	33%
$\sigma_{T}^{2}$	0.00	0%	0.09	0%
$\sigma_{I}^{2}$	15.74	3%	0.00	0%
$\sigma_{P*T}^{2}$	105.42	24%	117.01	29%
$\sigma_{P*I}^{2}$	51.94	12%	36.74	9%
$\sigma_{T*I}^{2}$	0.79	0%	0.38	0%
$\sigma_{\text{Residuals}}^{2}$	164.63	37%	120.63	29%
GT reliability estimates				
$R_{\mathrm{KRn}}$	0.98		0.99	
$R_{\mathrm{Cn}}$	0.52		0.67	

$\sigma^{2}$ = variance component, P = person, T = time, I = item; $R_{\mathrm{KRn}}$ = nested between-person reliability; $R_{\mathrm{Cn}}$ = nested within-person reliability.

The stability estimates were high with *r* = 0.86 for interoceptive intensity and *r* = 0.91 for interoceptive valence (see Supplementary Figure 1).

#### Analyses for between person validity

Interoceptive intensity and valence were negatively associated with each other with a comparable effect size as in study 1 (see Table 6). The MAIA-2 sum score was associated with the MIVS but not the MIIS at Level 2. No single subscale of the MAIA-2 was significantly associated with MIIS, but we found positive associations with attention regulation and trusting for the MIVS (see Supplementary Table 5). Furthermore, most subscales were positively associated with the body awareness item at the trait level but not with Trusting and Not-Worrying. The MIIS was associated with the aggregated awareness item, but the MIVS was not, suggesting that the general awareness of bodily sensations captured intensity rather than valence of interoception. Furthermore, the MIIS was significantly and negatively associated with resilience (CD-RISC) and positively with anxiety sensitivity (ASI). The MIVS was positively associated with safety (NPSS) and resilience (CD-RISC), and negatively with depression (STADI), anxiety sensitivity (ASI), and Muscle Dysmorphic Disorder (MDDI). However, the trait-level of the awareness item (BAW) did not show similar significant relationships.

**Table 6:** Pearson correlations between interoceptive intensity (MIIS) and interoceptive valence (MIVS) at the between-person Level 2 with all relevant between person variables.

Variable	1	2	3	4	5	6	7	8	9	10	11	12
1. MIIS	–	[−0.70, −0.45]	[0.13, 0.47]	[−0.27, 0.10]	[−0.09, 0.28]	[−0.23, 0.14]	[−0.18, 0.19]	[−0.34, 0.02]	[−0.06, 0.30]	[−0.42, −0.08]	[0.01, 0.36]	[−0.09, 0.27]
2. MIVS	−0.59 (< 0.001)	–	[−0.14, 0.23]	[0.07, 0.42]	[−0.29, 0.08]	[0.04, 0.39]	[−0.28, 0.08]	[0.11, 0.45]	[−0.43, −0.08]	[0.11, 0.45]	[−0.40, −0.05]	[−0.51, −0.19]
3. BAW	0.31 (< 0.001)	0.05 (0.601)	–	[−0.01, 0.35]	[−0.20, 0.17]	[0.28, 0.58]	[−0.14, 0.23]	[−0.10, 0.27]	[−0.33, 0.03]	[−0.02, 0.34]	[−0.25, 0.11]	[−0.28, 0.08]
4. Age	−0.09 (0.349)	0.26 (0.006)	0.18 (0.060)	–	[−0.03, 0.33]	[0.13, 0.47]	[−0.30, 0.06]	[0.02, 0.38]	[−0.48, −0.15]	[0.23, 0.54]	[−0.44, −0.09]	[−0.37, −0.02]
5. Gender	0.10 (0.316)	−0.11 (0.256)	−0.01 (0.897)	0.16 (0.097)	–	[−0.35, 0.01]	[−0.45, −0.11]	[−0.22, 0.15]	[−0.18, 0.19]	[−0.22, 0.15]	[−0.17, 0.20]	[−0.07, 0.30]
6. MAIA	−0.05 (0.627)	0.22 (0.019)	0.44 (< 0.001)	0.31 (< 0.001)	−0.17 (0.067)	-	[−0.18, 0.18]	[0.21, 0.53]	[−0.58, −0.28]	[0.39, 0.66]	[−0.56, −0.26]	[−0.47, −0.13]
7. HSPS	0.00 (0.958)	−0.10 (0.281)	0.05 (0.601)	−0.12 (0.192)	−0.29 (0.002)	0.00 (0.999)	–	[−0.23, 0.14]	[0.14, 0.48]	[−0.35, 0.01]	[0.20, 0.52]	[−0.03, 0.33]
8. NPSS	−0.17 (0.075)	0.29 (0.002)	0.08 (0.374)	0.21 (0.028)	−0.04 (0.695)	0.38 (< 0.001)	−0.05 (0.612)	–	[−0.67, −0.41]	[0.32, 0.61]	[−0.43, −0.09]	[−0.55, −0.23]
9. STADI	0.13 (0.180)	−0.26 (0.005)	−0.15 (0.104)	−0.32 (< 0.001)	0.00 (0.967)	−0.45 (< 0.001)	0.32 (< 0.001)	−0.55 (< 0.001)	–	[−0.79, −0.60]	[0.29, 0.59]	[0.27, 0.57]
10. CD-RISC	−0.26 (0.006)	0.29 (0.002)	0.16 (0.085)	0.40 (< 0.001)	−0.04 (0.681)	0.54 (< 0.001)	−0.18 (0.060)	0.48 (< 0.001)	−0.70 (< 0.001)	–	[−0.56, −0.26]	[−0.47, −0.13]
11. ASI	0.19 (0.041)	−0.23 (0.013)	−0.07 (0.441)	−0.27 (0.003)	0.02 (0.861)	−0.42 (< 0.001)	0.37 (< 0.001)	−0.27 (0.004)	0.45 (< 0.001)	−0.42 (< 0.001)	–	[0.14, 0.47]
12. MDDI	0.09 (0.325)	−0.36 (< 0.001)	−0.10 (0.278)	−0.20 (0.032)	0.12 (0.201)	−0.31 (< 0.001)	0.15 (0.110)	−0.40 (< 0.001)	0.43 (< 0.001)	−0.31 (< 0.001)	0.32 (< 0.001)	–

#### Within-person validity

##### Associations between interoceptive intensity, valence, and body awareness

As in study 1, valence ratings significantly and negatively predicted intensity at both Level 1 and Level 2 explaining about 25% of the variance in the MIIS (see Table 7). When adding quadratic terms, the negative quadratic effect of situational valence was significant, again indicating a curvilinear relationship where the negative association between valence and intensity is stronger at higher levels of valence (see Supplementary Table 6).

**Table 7:** Interoceptive intensity predicted via interoceptive valence at Level 1 and Level 2.

Predictors	Estimates	Conf. Int (95%)	*p*-value
Intercept	71.37	61.47–81.27	**<0.001**
MIVS (Level 2)	−0.53	−0.68 – −0.39	**<0.001**
MIVS (Level 1)	−0.43	−0.48 – −0.37	**<0.001**
Random effects			
σ^2^	122.94		
τ_00 Participant_	85.62		
τ_11 Participant. MIVS (Level 1)_	0.06		
ρ_01 Participant_	−0.06		
ICC	0.44		
N _Participant_	112		
Observations	5,503		
Marginal *R*^2^/Conditional *R*^2^	0.248/0.577		

MIVS, Momentary interoceptive valence scale.

As illustrated in Table 8, the BAW item was positively associated with interoceptive intensity at both Level 1 and Level 2 and negatively with valence at Level 1. The pleasure and arousal ratings showed the same pattern as in study 1. Furthermore, perceived safety as well as perceived loudness of the surroundings were associated with both variables at Level 1. Specifically, feeling safe was associated with a lower perceived intensity and more positive valence of bodily signals. Louder environments went along with more intense interoceptive sensations and more negative valence.

**Table 8:** Interoceptive intensity (MIIS) and interoceptive valence (MIVS) predicted via various variables at Level 1 and Level 2 indicating validity.

Predictors	MIIS			MIVS		
	Estimates	Conf. Int (95%)	*p*-value	Estimates	Conf. Int (95%)	*p*-value
Intercept	23.55	−2.20 – 49.29	0.073	46.03	17.98–74.08	**0.001**
BAW (Level 2)	2.92	1.56–4.29	**<0.001**	0.52	−0.97 – 2.01	0.491
BAW (Level 1)	2.82	2.53–3.12	**<0.001**	−1.49	−1.79 – −1.19	**<0.001**
Pleasure (Level 2)	0.80	−3.84 – 5.43	0.734	3.46	−1.59 – 8.51	0.177
Pleasure (Level 1)	−1.64	−2.11 – −1.17	**<0.001**	2.77	2.30–3.24	**<0.001**
Arousal (Level 2)	2.81	−0.68 – 6.31	0.114	−4.92	−8.73 – −1.11	**0.012**
Arousal (Level 1)	2.24	1.82–2.66	**<0.001**	−2.60	−3.02 – −2.18	**<0.001**
Dominance (Level 2)	−0.59	−3.28 – 2.09	0.663	0.12	−2.80 – 3.05	0.933
Dominance (Level 1)	1.35	0.88–1.83	**<0.001**	−0.09	−0.57 – 0.38	0.701
Safety (Level 2)	−4.53	−8.76 – −0.29	**0.037**	5.21	0.59–9.82	**0.027**
Safety (Level 1)	−0.73	−1.32 – −0.14	**0.015**	1.87	1.29–2.46	**<0.001**
Loudness (Level 2)	0.31	0.15–0.48	**<0.001**	-0.14	−0.32 – 0.04	0.129
Loudness (Level 1)	0.04	0.03–0.06	**<0.001**	−0.04	−0.06 – −0.02	**<0.001**
Location (at home)	1.77	0.96–2.58	**<0.001**	−0.82	−1.63 – −0.02	**0.045**
Alone vs. in contact	0.25	−0.50 – 1.01	0.511	0.13	−0.62 – 0.88	0.738
Random effects						
σ^2^	140.64			138.57		
τ_00_	87.59 _Participant_			104.85 _Participant_		
ICC	0.38			0.43		
*N*	112 _Participant_			112 _Participant_		
Observations	5,503			5,503		
Marginal *R*^2^/Conditional *R*^2^	0.220/0.519			0.214/0.552		

BAW, Body awareness item.

When additionally fitting the multilevel models separately for each of the three MIIS items (see Supplementary Table 7) we found a relatively homogeneous pattern, with most effects in the same direction. However, safety at level 1 and the context variable location was not significant for gut but for breath/heart. This again suggests that the observed effects for the sum score seem context dependent to some degree.

### Discussion study 2

Study 2 replicated the reliability estimates of study 1 and found a similar pattern of associations, further supporting the validity of the MIIS and MIVS at both the between- and within-person levels. As an extension of study 1, we additionally examined between-person relationships with an established questionnaire, the MAIA-2 (Mehling et al., 2018), as well as with a single ambulatory item used in previous studies to assess body awareness in everyday-life situations (MacVittie et al., 2025; MacVittie et al., 2024).

Notably, at the between-person level, we found that the MAIA-2 was not linked with the interoceptive intensity (MIIS) but with the valence measure (MIVS). The MAIA was also positively associated with the person mean of the momentary body awareness item (MacVittie et al., 2025). In turn, this body awareness item correlated positively with the MIIS at the between-person level (but not with the MIVS). This might suggest that some aspects of body awareness at between-person level are related to interoceptive intensity and others are more strongly related to valence. These null findings for the MIIS likely reflect a well-documented trait–state divergence in interoceptive assessment. Trait questionnaires, such as the MAIA, primarily capture generalized beliefs and self-concepts regarding bodily focus, which often fail to align with real-time measures in naturalistic contexts (Murphy et al., 2019). The lack of correlation with trait scales suggests that momentary interoceptive attention is a distinct phenomenon that cannot be assessed by retrospective questionnaires. However, trusting seems highly relevant for interoceptive valence ratings since it measures positive aspects such as “I feel my body is a safe place.” This highlights the validity of the ambulatory assessment. Beyond that, and in contrast to study 1, the MIIS showed a significant finding with lower resilience, higher anxiety sensitivity, and higher mean body awareness ratings. This adds evidence for the validity of the MIIS at the between-person level.

The compelling associations of between-person questionnaires with both MIIS and MIVS were not observed for the aggregated body awareness item assessed in the field. This pattern of findings further indicates the importance of assessing interoceptive intensity and valence more fine-grained and across multiple domains such as heart, breath, and gut, rather than relying on a single global awareness item alone (MacVittie et al., 2025). At the between-person level, the MIVS was additionally and negatively associated with depressive symptoms, anxiety sensitivity, and muscle dysmorphia, which confirms a link with these health-relevant psychological concept and aspects of interoception (Grunewald et al., 2023; May and Tapert, 2025). Of note, the Highly Sensitive Person Scale showed no link with any measure of interoception, which might signal that highly sensitive individuals are more strongly characterized by deviations in exteroceptive processing than interoceptive body sensations.

At the within-person level, moments with higher bodily awareness (assessed with the single item) were linked with higher intensity and lower valence of interoceptive sensations. This is in line with the findings of MacVittie et al. (2025), who reported higher arousal and lower emotional valence in moments with higher body awareness (see also Poerio et al., 2024). Furthermore, moments with higher perceived loudness were associated with more intense bodily sensations and with lower valence of these sensations. Thus, in louder or more arousing environments, people felt their bodies more intense and experienced these sensations as less pleasant. This might signal that people are more sensitive to internal as well as external sensory perceptions at similar moments and situations in everyday life, a pattern which might signal that similar processes such as heightened attention are relevant for both interoception and exteroception to a certain extent (Murphy et al., 2020; Pennebaker and Lightner, 1980; Lopez-Martin et al., 2025). The opposite pattern emerged for moments with more perceived safety in everyday life, where people perceived their body less intense and more positive. This is in accordance with EMA studies indicating states of lower physiological arousal (i.e., lower heart rate; higher heart rate variability) when feeling psychologically safe (Schwerdtfeger et al., 2022; Schwerdtfeger and Rominger, 2024b; Schwerdtfeger et al., 2025). Consequently, the lack of strong bodily sensations might signal momentary safety in everyday life.

However, the single-item analyses of the MIIS (see Supplementary Table 4) showed that this effect seems to rely most strongly on sensations of the breath and the heart, and we observed a reversed effect for the gut intensity item, although with a smaller effect size. This highlights that, for specific research questions, separate analyses of the intensity items may be preferable to using the total score of the MIIS or the MIVS. The composite approach ensures the scales capture systemic bodily focus (in terms of attention and appraisal) rather than isolated, localized somatic symptoms (e.g., breathlessness due to physical exertion). Empirical support for this aggregation replicated across both studies (Vlemincx et al., 2023; see Supplementary Tables 1, 2). Robust between-person inter-item correlations support a generalized tendency for somatic focus. Meanwhile, expectedly lower within-person correlations between breath/heart and gut items could be interpreted in terms of divergent physiological trajectories, underscoring the necessity of sampling across different organs to capture a more systemic state. In sum, these findings support the situational sensitivity and ecological validity of the MIIS and MIVS, respectively, demonstrating that both measures respond meaningfully and differently to variations in environmental contexts. Nevertheless, depending on specific research questions the application of single items of the MIIS and MIVS might be more adequate than using the total scale.

## General discussion

By applying two independent and well-powered EMA studies, we could show that the assessment of the perception of the intensity and valence of bodily signals can be accomplished reliably and validly, thus offering a feasible toolkit for the ambulatory assessment of interoceptive attention and appraisal as two important dimensions (Farb et al., 2015; Murphy et al., 2020; Khalsa et al., 2018). Figure 2 summarizes the core finding that interoceptive evaluations of bodily sensations (higher MIVS) seem to be associated with better well-being at the between-person level (Level 2), while intensity (MIIS) was not. When it comes to the within-person level (Level 1) both MIIS and MIVS showed comparable characteristics (although with opposing directions). In general, the findings call for a more fine-grained approach to assess interoceptive attention and appraisal in everyday life, thereby focusing on both the intensity of the perceived bodily signals and the valence.

**Figure 2:**
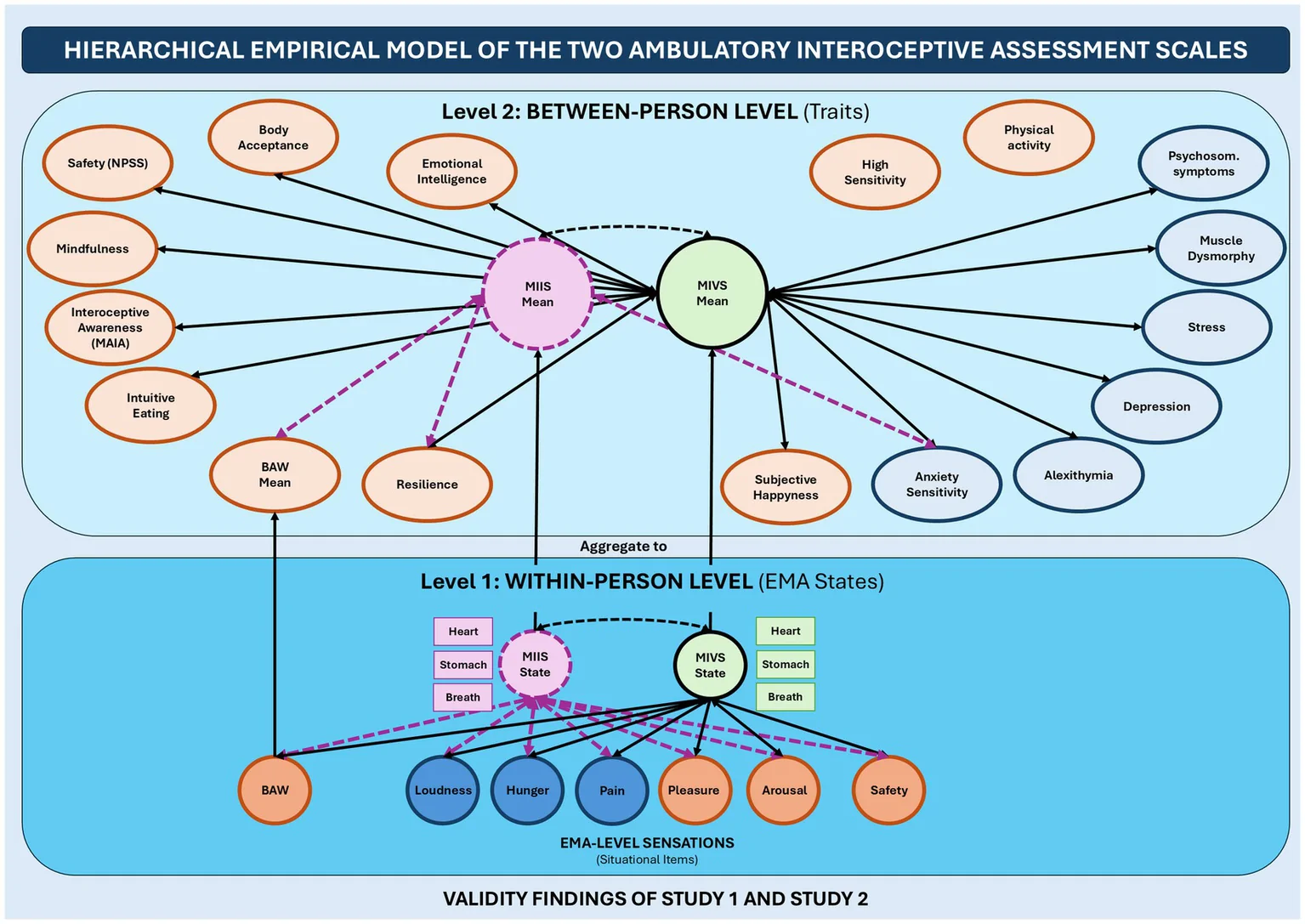
Illustration of main validity findings of study 1 and study 2.

Although promising results could be obtained, the proposed approach is not without limitations. First, although interoception is often conceptualized as comprising both conscious and unconscious processes (Nord and Garfinkel, 2022), the present work primarily focused on consciously perceived (and explicitly reported!) bodily sensations. Consequently, we cannot draw conclusions with respect to unconsciously perceived sensations (for similar argumentation, see Poerio et al., 2024). Second, our EMA design is observational and thus does not allow for causal conclusions. While we demonstrate systematic associations between interoceptive intensity and valence on the one hand, and a variety of psychological and situational variables on the other hand, the causal path of these relationships remains unclear. Hence, whether interoception drives the effects or is influenced by them, or whether third hidden variables are causally linked with these variables, remains to be studied. Future research could make use of micro-randomized trials, experimental manipulations embedded in EMA protocols, or quasi-experimental designs to move closer to identifying mechanistic pathways of interoception (see, e.g., Klasnja et al., 2019; Rominger et al., 2026). Third, although we assessed interoceptive sensations across three core domains (heart, gut, and breath), the dimensionality of interoceptive experience is certainly broader, also covering proprioception and thermal perception, among other domains (Poerio et al., 2024; Nord and Garfinkel, 2022). Hence, refining the measures to account for a holistic perspective on interoception (e.g., via extending the item pool) could advance the field. In light of this, the within-person reliability indicators for the MIIS (0.42 and 0.52) were at the lower boundary, suggesting that the scale might benefit from additional items. Finally, although the samples of both studies were quite large, the compliance was at the lower end, and most participants were young students and did not represent the broader population. Replication in more heterogeneous samples, including clinical populations and different age groups, would help to clarify the generalizability of our findings and determine whether the observed patterns hold across different demographic and clinical contexts.

A further limitation is the shared methods variance, because all variables were subjective and we did not have objective performance measures included. Hence, effects sizes might have been inflated. Noteworthy, methods to assess interoceptive (cardiac) accuracy in the field are available and therefore future studies should extend this with respect to more objective indicators to further elaborate on the validity (e.g., Rominger and Schwerdtfeger, 2023; Höller et al., 2021; Ponzo et al., 2021). Nevertheless, it is important to note that we have assessed all trait questionnaires and the ambulatory questionnaires in very different situations throughout everyday life, which reduces the amount of shared contextual variance. This also highlights the strength of the method, which allows us to assess interoceptive intensity and valence at random moments and largely different situations. We believe that the assessment of interoceptive intensity and valence at the very moment *in situ* is an ecologically valid measure of interoceptive sensations. Assessing interoception at the very moment constitutes the most direct and immediate access to this subjective phenomenon. By obtaining multiple answers across multiple situations, we gain deeper insight into how and why interoceptive intensity and valence develop.

## Conclusion

Irrespective of the above-mentioned limitations, within two independent samples we could demonstrate that a momentary assessment of subjective interoceptive intensity and valence is reliable and valid, although reliability is at the lower boundary for interoceptive attention at Level 1. Both studies showed that interoceptive intensity and interoceptive valence are inversely related to each other and systematically linked to key validity constructs such as anxiety sensitivity, perceived stress, depression, mindfulness, and psychological safety. Relationships with gender, age, and physical activity were limited, while higher intensity of interoceptive sensations seemed, on average, associated with less pleasant and less positive valence, both in general (between-person) and in specific situations (within-person). In other words, stronger interoceptive sensations tend to be perceived as more negative, whereas more positive evaluations of bodily sensations (higher interoceptive valence) are associated with more positive affective well-being and fewer health-related complaints (see Figure 2). This indicates that the MIVS (and the MIIS) shares theoretical variance with general affective states. However, beyond that, the MIVS also provides unique utility. Our analyses showed that pain, hunger, and safety predicted the MIVS score independent of general affect, which confirms that the MIVS captures specific, incremental information about interoception.

These results support the idea that the valence of interoceptive sensations, rather than the attention to interoceptive signals (i.e., intensity) alone, may be important to understanding the beneficial effects of interoception in everyday life. Methodologically, we provide psychometric evaluations of an easy-to-use ambulatory solution to assess both attention and appraisal/valence of interoception on a momentary level, thus enabling field studies to capture both within- and between-person fluctuations of visceral sensations. Because both ambulatory questionnaires are brief and suitable for repeated administration, this novel method offers a scalable basis for research, moving beyond one single domain of visceral sensations. Future studies might combine it with the use of smartphones and wearables in large and diverse samples to investigate interoception more deeply. As such, the MIIS and MIVS offer promising tools for future research on the role of interoception in emotion, health, and well-being, and might open new avenues for studying how everyday interoceptive perceptions contribute to psychological functioning in real-world environments (Poerio et al., 2024; MacVittie et al., 2025).

## Data Availability

The raw data supporting the conclusions of this article will be made available by the authors, without undue reservation.
