# Supplementary material for: Do you feel your body right now? Ecological momentary assessment of interoceptive intensity and valence across multiple domains in everyday life
Source: Front Psychol. 2026 Oct 14;17:1930018. doi: 10.3389/fpsyg.2026.1930018 (PMC13713450; doi:10.3389/fpsyg.2026.1930018)
Supplement: Supplementary file 1 [file Supplementary_file_1.docx]

Supplemental Table 1. Correlation matrix of all three items of the MIIS

|  | **Study 1** | | | **Study 2** | | |
| --- | --- | --- | --- | --- | --- | --- |
| **Variable** | **1** | **2** | **3** | **1** | **2** | **3** |
| **1. Heart** | - | .86 (< .001) | .51 (< .001) | - | .89 (< .001) | .58 (< .001) |
| **2. Breath** | .61 (< .001) | - | .56 (< .001) | .68 (< .001) | - | .55 (< .001) |
| **3. Stomach/Gut** | .15 (< .001) | .16 (< .001) | - | .24 (< .001) | .24 (< .001) | - |

Note. Between-person correlations (based on aggregated person-means) are presented in the upper diagonal. Within-person correlations (repeated measures correlations) are presented in the lower diagonal. Exact p-values are reported in parentheses.

Supplemental Table 2. Correlation matrix of all three items of the MIVS

|  | **Study 1** | | | **Study 2** | | |
| --- | --- | --- | --- | --- | --- | --- |
| **Variable** | **1** | **2** | **3** | **1** | **2** | **3** |
| **1. Heart** | - | .94 (< .001) | .78 (< .001) | - | .93 (< .001) | .70 (< .001) |
| **2. Breath** | .67 ( < .001) | - | .80 ( < .001) | .75 (< .001) | - | .71 (< .001) |
| **3. Stomach/Gut** | .29 ( < .001) | .32 (< .001) | - | .38 (< .001) | .39 (< .001) | - |

Note. Between-person correlations (based on aggregated person-means) are presented in the upper diagonal. Within-person correlations (repeated measures correlations) are presented in the lower diagonal. Exact p-values are reported in parentheses.

Supplemental Table 3. Interoceptive intensity (MIIS) predicted via interoceptive valence (MIVS) at Level 1 and Level 2 as well as their quadratic terms.

| *Predictors* | *Estimates* | *Conf. Int (95%)* | *p-value* |
| --- | --- | --- | --- |
| Intercept | 33.35 | -4.62 – 71.31 | 0.085 |
| MIVS trait (Level 2) | 0.66 | -0.50 – 1.82 | 0.264 |
| MIVS trait quadratic (Level 2) | -0.01 | -0.02 – 0.00 | 0.058 |
| MIVS state (Level 1) | -0.45 | -0.48 – -0.42 | **<0.001** |
| MIVS state quadratic (Level 1) | -0.00 | -0.01 – -0.00 | **<0.001** |
| **Random Effects** | | | |
| σ^2^ | 144.44 | | |
| τ_00_ _Participant_ | 100.98 | | |
| ICC | 0.41 | | |
| N _Participant_ | 141 | | |
| Observations | 5907 | | |
| Marginal R^2^ / Conditional R^2^ | 0.227 / 0.545 | | |

Supplemental Table 4. Multilevel model predicting interoceptive intensity items (heart, breath, gut) with relevant variables at Level 1 and Level 2.

|  | **Heart** | | | **Breath** | | | **Gut** | | |
| --- | --- | --- | --- | --- | --- | --- | --- | --- | --- |
| *Predictors* | *Estimates* | *Conf. Int (95%)* | *p-value* | *Estimates* | *Conf. Int (95%)* | *p-value* | *Estimates* | *Conf. Int (95%)* | *p-value* |
| Intercept | 7.71 | -14.38 – 29.79 | 0.494 | 29.47 | 7.12 – 51.82 | **0.010** | 34.23 | 11.40 – 57.07 | **0.003** |
| Pleasure (Level 2) | 1.00 | -3.39 – 5.39 | 0.652 | -1.15 | -5.59 – 3.30 | 0.611 | -3.92 | -8.46 – 0.62 | 0.090 |
| Pleasure (Level 1) | -1.23 | -1.82 – -0.64 | **<0.001** | -0.60 | -1.18 – -0.03 | **0.040** | -0.73 | -1.38 – -0.08 | **0.029** |
| Arousal (Level 2) | 5.98 | 2.37 – 9.60 | **0.001** | 4.76 | 1.09 – 8.42 | **0.011** | 4.01 | 0.28 – 7.75 | **0.035** |
| Arousal (Level 1) | 4.93 | 4.41 – 5.45 | **<0.001** | 3.24 | 2.73 – 3.74 | **<0.001** | 0.65 | 0.08 – 1.23 | **0.026** |
| Dominance (Level 2) | 1.39 | -2.04 – 4.82 | 0.425 | -0.03 | -3.50 – 3.45 | 0.988 | -2.55 | -6.10 – 1.00 | 0.157 |
| Dominance (Level 1) | 1.81 | 1.18 – 2.43 | **<0.001** | 0.98 | 0.37 – 1.58 | **0.002** | 0.39 | -0.30 – 1.07 | 0.273 |
| Hunger (Level 2) | 0.38 | 0.19 – 0.56 | **<0.001** | 0.38 | 0.20 – 0.57 | **<0.001** | 0.40 | 0.21 – 0.59 | **<0.001** |
| Hunger (Level 1) | -0.01 | -0.03 – 0.01 | 0.281 | 0.01 | -0.01 – 0.03 | 0.433 | 0.14 | 0.12 – 0.17 | **<0.001** |
| Pain (Level 2) | -0.16 | -0.37 – 0.06 | 0.153 | -0.25 | -0.47 – -0.03 | **0.025** | 0.06 | -0.16 – 0.28 | 0.590 |
| Pain (Level 1) | 0.05 | -0.00 – 0.09 | 0.067 | 0.05 | -0.00 – 0.09 | 0.050 | 0.25 | 0.20 – 0.30 | **<0.001** |
| Location (at home) | -1.15 | -2.21 – -0.09 | **0.033** | -2.70 | -3.74 – -1.65 | **<0.001** | 2.67 | 1.52 – 3.82 | **<0.001** |
| Alone vs. in contact | 0.68 | -0.37 – 1.72 | 0.203 | 1.38 | 0.36 – 2.40 | **0.008** | -1.90 | -3.04 – -0.75 | **0.001** |
| **Random Effects** | | | | | | | | | |
| σ^2^ | 285.72 | | | 274.63 | | | 353.22 | | |
| τ_00_ | 148.49 _Participant_ | | | 152.58 _Participant_ | | | 158.29 _Participant_ | | |
| ICC | 0.34 | | | 0.36 | | | 0.31 | | |
| N | 141 _Participant_ | | | 141 _Participant_ | | | 141 _Participant_ | | |
| Observations | 5905 | | | 5905 | | | 5905 | | |
| Marginal R^2^ / Conditional R^2^ | 0.109 / 0.414 | | | 0.086 / 0.412 | | | 0.110 / 0.385 | | |

Supplemental Figure 1. Split-half stability of MIIS (interoceptive intensity, left) and MIVS (valence ratings, right). Slope and corresponding credibility intervals are illustrated.

Supplemental Table 5. Pearson correlations between interoceptive intensity (MIIS) and interoceptive valence (MIVS) at the between-person Level 2 with all subscales of the MAIA-2.

| **Variable** | **1** | **2** | **3** | **4** | **5** | **6** | **7** | **8** | **9** | **10** | **11** |
| --- | --- | --- | --- | --- | --- | --- | --- | --- | --- | --- | --- |
| **1. MIIS** | - | [-.68, -.43] | [.10, .45] | [-.14, .23] | [-.06, .31] | [-.29, .08] | [-.21, .16] | [-.13, .24] | [-.17, .20] | [-.19, .19] | [-.34, .02] |
| **2. MIVS** | -.57 (< .001) | - | [-.09, .27] | [-.06, .30] | [-.25, .12] | [-.07, .29] | [.05, .40] | [-.02, .34] | [-.01, .35] | [-.12, .25] | [.04, .40] |
| **3. BAW** | .28 (.002) | .09 (.329) | - | [.10, .44] | [.06, .41] | [-.22, .16] | [.19, .52] | [.19, .51] | [.23, .54] | [.25, .56] | [-.01, .35] |
| **4. Noticing** | .05 (.610) | .12 (.199) | .28 (.003) | - | [.01, .37] | [-.25, .12] | [.18, .50] | [.51, .74] | [.25, .56] | [.20, .52] | [.02, .38] |
| **5. Non-distracting** | .13 (.167) | -.07 (.479) | .24 (.009) | .20 (.039) | - | [-.04, .33] | [-.12, .25] | [.02, .38] | [-.04, .32] | [.21, .53] | [-.17, .20] |
| **6. Not-worrying** | -.11 (.268) | .11 (.230) | -.03 (.744) | -.06 (.508) | .15 (.118) | - | [-.15, .22] | [-.25, .12] | [-.21, .16] | [-.20, .17] | [-.28, .09] |
| **7. Attention regulation** | -.03 (.764) | .23 (.015) | .37 (< .001) | .35 (< .001) | .07 (.475) | .04 (.702) | - | [.22, .53] | [.38, .65] | [.28, .58] | [.31, .60] |
| **8. Emotional awareness** | .06 (.540) | .17 (.079) | .36 (< .001) | .64 (< .001) | .21 (.030) | -.06 (.501) | .39 (< .001) | - | [.35, .63] | [.33, .62] | [.03, .38] |
| **9. Self-regulation** | .01 (.892) | .18 (.063) | .39 (< .001) | .41 (< .001) | .14 (.128) | -.03 (.758) | .53 (< .001) | .50 (< .001) | - | [.33, .62] | [.27, .58] |
| **10. Body listening** | .00 (.998) | .06 (.497) | .42 (< .001) | .37 (< .001) | .39 (< .001) | -.01 (.899) | .44 (< .001) | .49 (< .001) | .49 (< .001) | - | [.22, .53] |
| **11. Trusting** | -.17 (.082) | .23 (.016) | .18 (.065) | .21 (.029) | .01 (.879) | -.10 (.284) | .47 (< .001) | .21 (.023) | .44 (< .001) | .39 (< .001) | - |

Note. MIIS (Momentary interoceptive intensity scale), MIVS (Momentary interoceptive valence scale), BAW (Body Awareness), MAIA (Multidimensional Assessment of Interoceptive Awareness). Exact p-values are in parentheses, and 95% confidence intervals are reported for all correlations in the upper diagonal

Supplemental Table 6. Interoceptive intensity (MIIS) predicted via interoceptive valence (MIVS) at Level 1 and Level 2 as well as their quadratic terms for study 2.

| *Predictors* | *Estimates* | *Conf. Int (95%)* | *p-value* |
| --- | --- | --- | --- |
| Intercept | 59.57 | 14.61 – 104.53 | **0.009** |
| MIVS trait (Level 2) | -0.18 | -1.51 – 1.16 | 0.792 |
| MIVS trait quadratic (Level 2) | -0.00 | -0.01 – 0.01 | 0.609 |
| MIVS state (Level 1) | -0.48 | -0.51 – -0.45 | **<0.001** |
| MIVS state quadratic (Level 1) | -0.00 | -0.00 – -0.00 | **<0.001** |
| **Random Effects** | | | |
| σ^2^ | 130.11 | | |
| τ_00_ _Participant_ | 84.31 | | |
| ICC | 0.39 | | |
| N _Participant_ | 112 | | |
| Observations | 5503 | | |
| Marginal R^2^ / Conditional R^2^ | 0.256 / 0.549 | | |

Supplemental Table 7. Multilevel model predicting interoceptive intensity items (heart, breath, gut) with relevant variables at Level 1 and Level 2 (study 2).

|  | **Heart** | | | **Breath** | | | **Gut** | | |
| --- | --- | --- | --- | --- | --- | --- | --- | --- | --- |
| *Predictors* | *Estimates* | *Conf. Int (95%)* | *p-value* | *Estimates* | *Conf. Int (95%)* | *p-value* | *Estimates* | *Conf. Int (95%)* | *p-value* |
| Intercept | 25.39 | -2.13 – 52.91 | 0.071 | 30.93 | 2.79 – 59.08 | **0.031** | 14.50 | -20.94 – 49.95 | 0.422 |
| BAW (Level 2) | 3.20 | 1.74 – 4.66 | **<0.001** | 3.16 | 1.68 – 4.63 | **<0.001** | 2.45 | 0.57 – 4.33 | **0.011** |
| BAW (Level 1) | 3.36 | 2.97 – 3.75 | **<0.001** | 3.27 | 2.87 – 3.66 | **<0.001** | 1.65 | 1.24 – 2.05 | **<0.001** |
| Pleasure (Level 2) | 0.99 | -3.97 – 5.94 | 0.693 | -2.01 | -7.02 – 3.00 | 0.432 | 3.45 | -2.93 – 9.83 | 0.286 |
| Pleasure (Level 1) | -1.57 | -2.19 – -0.95 | **<0.001** | -1.52 | -2.15 – -0.88 | **<0.001** | -2.18 | -2.82 – -1.54 | **<0.001** |
| Arousal (Level 2) | 3.05 | -0.68 – 6.79 | 0.108 | 2.35 | -1.43 – 6.12 | 0.223 | 2.94 | -1.87 – 7.75 | 0.228 |
| Arousal (Level 1) | 3.18 | 2.63 – 3.74 | **<0.001** | 2.22 | 1.66 – 2.79 | **<0.001** | 1.27 | 0.71 – 1.84 | **<0.001** |
| Dominance (Level 2) | -1.25 | -4.12 – 1.61 | 0.388 | -1.60 | -4.50 – 1.30 | 0.280 | 1.04 | -2.66 – 4.73 | 0.579 |
| Dominance (Level 1) | 1.75 | 1.12 – 2.37 | **<0.001** | 1.32 | 0.69 – 1.96 | **<0.001** | 0.69 | 0.05 – 1.33 | **0.034** |
| Safety (Level 2) | -4.82 | -9.36 – -0.28 | **0.038** | -2.89 | -7.48 – 1.70 | 0.217 | -5.94 | -11.77 – -0.10 | **0.046** |
| Safety (Level 1) | -1.80 | -2.56 – -1.03 | **<0.001** | -1.11 | -1.88 – -0.33 | **0.005** | 0.65 | -0.15 – 1.44 | 0.112 |
| Loudness (Level 2) | 0.31 | 0.13 – 0.49 | **0.001** | 0.35 | 0.17 – 0.53 | **<0.001** | 0.28 | 0.05 – 0.51 | **0.017** |
| Loudness (Level 1) | 0.06 | 0.04 – 0.09 | **<0.001** | 0.05 | 0.03 – 0.08 | **<0.001** | 0.03 | 0.01 – 0.06 | **0.011** |
| Location (at home) | 2.35 | 1.30 – 3.39 | **<0.001** | 2.66 | 1.62 – 3.71 | **<0.001** | 0.03 | -1.07 – 1.14 | 0.955 |
| Alone vs. in contact | 0.08 | -0.90 – 1.06 | 0.878 | 0.72 | -0.27 – 1.72 | 0.152 | -0.02 | -1.04 – 1.00 | 0.966 |
| **Random Effects** | | | | | | | | | |
| σ^2^ | 237.45 | | | 247.15 | | | 261.36 | | |
| τ_00_ | 98.25 _Participant_ | | | 103.55 _Participant_ | | | 165.63 _Participant_ | | |
| ICC | 0.29 | | | 0.30 | | | 0.39 | | |
| N | 112 _Participant_ | | | 112 _Participant_ | | | 112 _Participant_ | | |
| Observations | 5503 | | | 5503 | | | 5503 | | |
| Marginal R^2^ / Conditional R^2^ | 0.198 / 0.433 | | | 0.184 / 0.425 | | | 0.094 / 0.446 | | |
